# Supplementary material for: Functional diversity of rhizosphere soil microbial communities in response to different tillage and crop residue retention in a double-cropping rice field
Source: PLoS One. 2020 May 21;15(5):e0233642. doi: 10.1371/journal.pone.0233642 (PMC7241825; doi:10.1371/journal.pone.0233642)
Supplement: Attachment — Submitted filename: PLOSONE0303.doc [file pone.0233642.s001.doc]

**Title page**

**Title**

Functional diversity of rhizosphere soil microbial communities in response to different soil tillage and crop residue retention in a double-cropping rice field

**Author names**

Haiming Tang^*^, Chao Li, Xiaoping Xiao, Xiaochen Pan, Wenguang Tang, Kaikai Cheng, Ke Wang, Weiyan Li

**Affiliations**

Hunan Soil and Fertilizer Institute, Changsha 410125, China

*** Corresponding author**

Haiming Tang

Tel: +86 731 84696102; Fax: +86 731 84691581; E-Mail address: [tanghaiming66@163.com](mailto:tanghaiming66@163.com).

**Functional diversity of rhizosphere soil microbial communities in response to different soil tillage and crop residue retention in a double-cropping rice field**

**Abstract**: Microbial community functional diversity is a sensitive indicator of soil quality, soil managements such as soil tillage and crop residues which can affect the microbial community functional diversity of paddy fields. However, there is still limited information about the influence of different soil tillage and crop residues managements on the rhizosphere soil microbial community functional diversity in a double-cropping rice (*Oryza sativa* L.) fields. Therefore, the short-term effects of different soil tillage and incorporated residues managements on the average well color development (AWCD), richness and evenness of the functional diversity, and the principal component analysis (PCA) of substrate reaction were investigated in the present paper, in a double-cropping rice system of southern China. The experiment included four soil tillage treatments: conventional tillage with residue incorporation (CT), rotary tillage with residue incorporation (RT), no tillage with residue retention (NT), and rotary tillage with residue removed as control (RTO). The results showed that the values of AWCD with application of crop residues treatments were higher than that of the treatment without crop residues. It was implied that application of crop residues managements resulted in the variation of the carbon utilization efficiency of rhizosphere soil microbial communities. At maturity stages of early and late rice, the richness indices, Shannon indices and McIntosh indices with CT treatments were significantly higher than that of RTO treatment, and the order of was CT>RT>NT>RTO. PCA results indicated that there were significant differences in carbon substrate utilization patterns among different soil tillage treatments. Carbohydrates and amino acids were the main carbon resources utilized by rhizosphere microbes. Therefore, the combined application of soil tillage and crop residues managements could significantly increase the rhizosphere soil microbial community functional diversity in the double-cropping paddy field of southern China.

**Key words**: rice; soil tillage; residue management; rhizospheric soil; community functional diversity

**Introduction**

In recent years, the traditional practice of intensive cultivation involving several deep ploughings and complete removal of crop residues has resulted in soils low in organic matter, of poor fertility and very susceptible to erosion. Some studies indicated that the conservation agriculture has been found to increase crop yield, improve water use efficiency, reduce energy inputs, and improve soil fertility [1-2]. Retention of crop residues increases soil organic carbon (C) and nitrogen (N) stocks [3], and the reduction or elimination of tillage reduces soil respiration, resulting in more C in the soil [4-5].

In all ecosystems, soil microbes play key roles in the decomposition of organic matter, in nutrient cycling and in altering the availability of nutrients to plants [6]. The soil microbial community is closely related to the quantity and quality of carbon and nutrients from plant residues and organic inputs, so it has been used as a sensitive indicator to predict soil biological conditions and the effect of agricultural practice in soil ecosystem [7]. Understanding the impact of management practices on the soil microbe community structure and diversity is important for evaluating the effectiveness of a management regime [8]. Some studies indicated that both the absolute quantity and the composition and function of soil microbial communities have been shown to change under conservation agriculture [9-10].

The Yangtze River Plain is one of the most important rice production areas in China [11]. Chinese milk vetch (*Astragalus sinicus* L.) and early rice and late rice (double cropping rice) (*Oryza sativa* L.) are important crops in this region. Recently, the Chinese milk vetch and rice straw residues as organic fertilizer returning to the paddy soil were accepted by more and more people for that it provides favorable soil environment and nutrients for rice plant growth [12]. Therefore, the amount of application of N and P fertilizer has reduced under adoption of this practice conditions. Meanwhile, rotary tillage and no tillage with residues retention practices are becoming increasingly popular regionally. However, there is little information about the effects of different soil tillage and crop residues management practices on the rhizospheric soil microbial communities in the double-cropping rice systems of southern China. We hypothesized that the combined application of soil tillage and crop residues managements would affect the rhizosphere soil microbial community function in the double-cropping rice field.

Therefore, the aims of this study were: (1) to analyze the effect on rhizospheric soil microbial activity and function following 4 years of continuous application of conventional tillage, rotary tillage and no tillage with residue incorporation, and rotary tillage with residue removed, (2) to explore the main carbon resources utilized with different soil tillage treatments, and (3) to select an appropriate soil tillage practice for paddy fields in a milk vetch–double cropping rice system.

**Materials and methods**

**Study site**

The experiment was established in 2015. It was located in Ning Xiang County (28°07′ N, 112°18′ E) of Hunan Province, China. Under a continental monsoon climate, the annual mean precipitation and evapotranspiration were 1553 and 1354 mm, respectively. The monthly mean temperature was 17.2°C. The predominant soil at the experimental site was a Stagnic Anthrosols and it was developed from the Quaternary red earth (clay loam). The soil texture in the topsoil (0-20 cm) was silt clay loam with 13.85% sand and 56.64% silt. At the beginning of the study, the surface soil characteristics (0-20 cm) were as follows: soil organic carbon (SOC) 22.07 g kg^-1^, total nitrogen (N) 2.14 g kg^-1^, total phosphorous (P) 0.82 g kg^-1^, total potassium (K) 13.21 g kg^-1^, available N 192.20 mg kg^-1^, available P 13.49 mg kg^-1^, and available K 81.91 mg kg^-1^, pH 5.79. There were three crops in a year, Chinese milk vetch (*Astragalus sinicus* L.), early rice and late rice (*Oryza sativa* L.). Chinese milk vetch was sown at the end of October and returned to the paddy field in early April of the following year. Early rice was then transplanted and harvested in the middle of July. Late rice were transplanted in the middle of July and harvested in the end of October.

**Field experiment**

The four soil tillage treatments were initiated in 2015. The systems tested included: conventional tillage with residue incorporation (CT), rotary tillage with residue incorporation (RT), no tillage with residue retention (NT), rotary tillage with all residue removed as control (RTO). The dimensions of the plots were 56.0 m^2^ (7 m×8 m), and treatments were laid out in a randomized complete block design with three replications. Chinese milk vetch and rice straw residues were retained for the CT, RT and NT treatments after both the Chinese milk vetch, early and late rice crops were harvested. The quantity of Chinese milk vetch, early and late rice straw residues added into the paddy soil for the CT, RT and NT treatments were 22500, 2000, and 2000 kg hm^-2^, respectively. The quantity of Chinese milk vetch, early and late rice straw residues removed from the paddy soil for the CT, RT and NT treatments were 29500, 3400, and 4000 kg hm^-2^, respectively, and the quantity of Chinese milk vetch, early and late rice straw residues removed from the paddy soil for the RTO treatment were 52000, 5400, and 6000 kg hm^-2^, respectively. And the carbon content of Chinese milk vetch, early and late rice straw residues were 386.4 g kg^-1^, 395.3 g kg^-1^, and 400.5 g kg^-1^, respectively. No tillage management was adopted in the NT treatments, and the Chinese milk vetch and rice straw residues were mixed as covering crops on the soil surface before transplanting of rice seedlings. Irrigation water was keep at depth of 2 cm above soil surface with CT, RT and RTO treatments when taken soil tillage managements, then transplanting of rice seedlings. Chinese milk vetch and rice straw residues were incorporated into the soil with tillage managements under CT and RT treatments. The CT treatments were tilled once with a moldboard plow to a depth of 15–20 cm and then rotovated twice to a depth of 8–10 cm before transplanting the rice seedlings. The RT and RTO treatments were rotovated four times to a depth of 8–10 cm before transplanting the rice seedlings. The soil tillage practices with the RTO treatments were similar with that of the RT treatment except that Chinese milk vetch and rice straw residues were removed in both early rice and late rice seasons.

The early and late rice seedlings were manually transplanted to the paddy in April and July, and harvested with a combine in July and October, respectively. The cultivars of early rice were Xiangzaoxian 45 and the late rice were Xiangwanxian 13 in 2016, 2017 and 2018, respectively. One-month-old seedlings were transplanted at a density of 150,000 plants hm^-2^. The experiment ensured all treatments received the same amount of N, phosphorus pentoxide (P_2_O_5_), potassium oxide (K_2_O) (the total amount of N, P_2_O_5_, K_2_O in mineral fertilizer and that from Chinese milk vetch or rice straw residues) during the early and late rice growing season, respectively. The chemical fertilizer including urea, ordinary superphosphate and potassium chloride. For both early and late rice, the quantity of N were applied at the rate of 150.0 and 180.0 kg hm^-2^ (60% and 40% at basal and tillering stage), 75.0 kg hm^-2^ of P_2_O_5_ as superphosphate, and 120.0 kg hm^-2^ of K_2_O as potassium chloride. All the P and K fertilizers were applied at soil tillage before rice transplanting. Paraquat (1, 1′-dimethyl-4, 4′ bipyridiniumion) was applied with 6.0 kg hm^-2^ to control weeds under NT treatment and 1.5 kg hm^-2^ for RT, CT and RTO treatments before the early and late rice transplanting.

**Soil sampling**

Soil samples were collected at the maturity stages of early rice and late rice in the middle of late July, and October 2018. Rhizosphere soil was operationally defined as soil adhering to the total roots after gentle shaking. The whole plant with their roots were extracted from soil and after shaking off the loosely adhering soil, the tightly adhering soil (i.e. rhizosphere soil) was carefully collected. In order to obtain the enough rhizosphere soil for multiplicating, twenty plants were randomly selected from each plot, and these rhizosphere soils were pooled to form one composite sample. Thus, three composite samples of each soil tillage treatment were collected at sampling time, and a total of 12 composite samples were taken for early rice and late rice maturity stages, respectively. The fresh samples were placed immediately on ice and transported to the laboratory. Plant roots were removed by passing the samples through a 2-mm mesh sieve, and the soil samples were then stored at -20℃ until molecular analysis.

**Soil analysis**

The functional diversity of rhizosphere soil microorganisms were tested by using Biolog-Eco (Biolog Inc., Hayward, CA, USA) test plates, following a procedure adapted from Garland and Mills (1991) [13]. To use the same mass of soil samples for analysis, a certain quantity of fresh soil equivalent to 10.0 g of fresh soil was added to a flask containing 90 ml of sterile water, and then the flask was shaken for 20 min with a speed of 200 r/min after being sealed. Afterwards, the sterile water was diluted three times by the ten-time dilution method, and 125 μl of the diluted solution was inoculated in each tiny pore of a Biolog-Eco plate. Each treatment had one plate and repeated three times. The inoculated plates were cultured at 25 ℃ for 12 days, and absorbance was detected by Biolog ELX808 (Hayward, CA, USA) automatic disk reader at 590 nm every 12 h [3]. Data were transformed by using Mierolog 3.4.2 software (BiologInc.). Then the data within 120 h were used for principal component analysis.

**Statistical analysis**

The data were compared by using one-way analysis of variance (ANOVA) (α=5%, SAS 9.3 [14]). Average well color development (AWCD), diversity index (richness, Shannon and McIntosh indices), principal component analysis (PCA) and other characteristic parameters can be used as new variables extracted from original Biolog data to analyze its relationship with carbons sources of rhizosphere soil microbial diversity [3].

**Results**

**Average well color development**

The value of average well color development (AWCD) can be used as a valid indicator of overall activity of soil microorganisms [3]. The changing rate of AWCD reflects the ability of soil microorganisms to utilize carbon source. At the early rice and late rice maturity stages, AWCD increased with the prolongation of incubation time. There is no change in AWCD with different soil tillage treatments at the beginning of 24 h, but increased rapidly within 24-120 h, then increased slowly until the end of the experiment. The results indicated that the value of AWCD were rapid increased until 120 h with CT, RT and NT treatments, and the AWCD were ended at 120 h with RTO treatment (Fig. 1). Meanwhile the results indicated that the value of AWCD with CT, RT and NT treatments were higher than that of the RTO treatment.

**Genetic diversity indices of rhizospheric soil microbial communities**

Richness, Shannon and McIntosh indices were used to reflect the richness and evenness of rhizospheric soil microbial community species, respectively. At maturity stages of early and late rice, Richness indices and McIntosh indices with CT treatments were significantly higher than that of RTO treatment, and the order of was CT>RT>NT>RTO. And the Shannon index of CT, RT and NT treatments was significantly higher than that of RTO treatment (Table 1). The results showed that the Richness indices, Shannon indices and McIntosh indices were increased by application of crop residues managements, compared with treatments without crop residues.

**PCA of metabolic function of microbial community in rhizosphere soil**

The value of AWCD data measured after 120 h of incubation were standardized and analyzed by PCA [15]. At maturity stages of early and late rice, according to the principle that the number of extracted principal components requires the cumulative variance contribution rate to 85% [16], eight principal components were extracted, and the cumulative contribution rates were 84.26% and 86.28%, respectively. At maturity stage of early rice, the variance contribution rates of the first principal component (PC1) and the second principal component (PC2) were 34.37% and 15.58%, and the contribution rates of principal components from 3 to 8 were 8.65%, 7.36%, 5.88%, 4.54%, 4.02% and 3.86%, respectively. At maturity stage of late rice, the variance contribution rates of the PC1 and PC2 were 35.58% and 17.28%, and the contribution rates of principal components from 3 to 8 were 8.12%, 7.05%, 5.24%, 5.09%, 4.27% and 3.65%, respectively. Therefore, only the first two principal components were analyzed in this paper (Fig. 2).

The results showed that there were obvious differences on PC axis between different soil tillage treatments. RTO treatment were mainly distributes in the negative direction of PC1 axis, while CT and RT treatments were mainly distributes in the positive direction of PC1 axis. RTO treatment were distributed in the negative direction of PC2 axis, CT and RT treatments were distributed in the positive direction of PC2 axis, NT treatment were distributes in both positive and negative directions of PC2 axis (Fig. 2). Therefore, there were significantly differences in soil microbial communities between different soil tillage treatments.

The load values of 35 kinds of carbon sources on PC1 and PC2 were further analyzed. The higher of the load values, the more significant the effect of the corresponding carbon sources on the principal components. According to |r|> 0.5, there were 20 kinds of carbon sources contributing to PC1, including 3 kinds of carbohydrates, 9 kinds of amino acids, 3 kinds of carboxylic acids, 1 kind of nucleosides, 2 kinds of phenolic esters, 1 kind of amines and 1 kind of polymers. There were 18 kinds of carbon sources contributing to PC2, including 12 kinds of carbohydrates, 3 kinds of carboxylic acids, 2 kinds of nucleosides and 1 kind of polymer (Table 2). The results showed that the carbohydrates and amino acids were the main carbon sources influencing the difference between PC1 and PC2. Amino acid carbon sources have a larger proportion in PC1, while the carbohydrates sources have a larger proportion in PC2. The main carbon sources to distinguish different between soil tillage treatments is carbohydrates and amino acids.

The first 12 kinds of carbon sources with different soil tillage treatments were analyzed in the present paper. The results showed that the main utilization of rhizosphere soil microorganisms with different soil tillage treatments is carbohydrates, amino acids and small amount of other substances. But there were fewer similarities of main carbon sources utilization between the different soil tillage treatments indicating that the utilization of carbon sources by rhizosphere soil microorganisms were changed under different soil tillage conditions (Table 3).

**PCA of carbohydrates, amino acids, carboxylic acids, and nucleosides in rhizosphere soil**

L-Serine, D-Glucosaminic acid, and Uridine-5P-monophosphate are the main carbon sources of amino acids, carboxylic acids and nucleosides with CT treatment, respectively. For carbohydrates, there are many kinds of carbon sources, such as D-Mannitol, α-D-Glucose and L-Methyl-D-glucoside, which play a decisive role in CT treatment (Table 2). PCA of carbohydrates, amino acids, carboxylic acids, and nucleosides in rhizosphere soil showed that both the CT and RT treatments were in the carbon source intensive region (Fig. 3 A, B, C, D). However, the RTO treatment was not in the carbon source intensive region.

**Discussion**

In the present paper, retention of crop residues treatments significantly increased soil microbial activity and functional diversity compared to crop residues removal treatment, indicating that residue retention is the key to improve soil biological fertility on this double cropping paddy field soil rather than the elimination of soil tillage. The results provided a reasonable hypothesis that enhanced soil conditions for increased rhizosphere soil microbial community function contribute due to the application of crop residues practices [17]. This conclusion was further reinforced by the similar performance of the crop residues removal treatments with and without tillage, indicating that under these soils and management conditions tillage exerted significant influence on soil functionality microbial ecology.

AWCD is an important indicator of net soil microbial community activity reflecting the utilization ability of single C sources. High AWCD value with crop residues retention and soil tillage treatments indicated that this practice can increase total microbial activity in the rhizosphere soil under a double-cropping rice field of southern China. Crop residues retention with soil tillage treatments benefits the soil by increasing the soil environment, moderating moisture and temperature variation across seasons and has been reported favorable to microbial growth in rhizosphere soil [18-19]. The Shannon and McIntosh indices can provide an indication of the soil microbial biocommunity and biodiversity while the Richness indices provide insight into the diversity of microorganisms. It was found that all the three indices were increased by crop residues retention and soil tillage managements, suggesting that the structure of the rhizosphere soil microbial communities may be altered by these two managements.

Under taking the soil tillage conditions, retaining crop residues on the paddy soil significantly increases most C sources, reflecting organic C accumulation and the provision of increased substrate supply for microorganisms. Some studies have also shown that applying conservation soil tillage and crop residues management practices increased microbial diversity as a result of increased organic C from retaining residues [20]. Similarly, regular addition of SOM may increase background levels of microbial activity, increase nutrient cycling, decrease the concentrations of easily available nutrient sources and increase microbial diversity [21]. Availability of soil carbohydrates is a useful indicator of changes in SOM status [22], and it has been reported that carbohydrates may account up to 75% of the total organic C of the soils in some environments and is/was a key component of the C cycle [23]. As such, when the soil microbial community is provided with increased carbohydrates from crop residues retention, activity and functional diversity increases with adaptations for greater carbohydrate utilization, leading to increased C cycling and soil fertility.

The positional differences of various samples in the PCA of Biolog-ECO metabolic fingerprints were related to the ability of soil microorganisms to use C substrates [13]. Crop residues retention treatments were distributed on the positive axis compared to the crop residues removal treatment on the negative axis (Fig. 2), with the score coefficients being significantly different, which likely occurred as a result of crop residues retention greatly contributing to rhizosphere soil nutrients by microbial decomposition. This result supports the hypothesis that the application of crop residues would significantly impact rhizosphere soil microbial function consistent with Myers et al. (2001) [24], who stated that differences in microbial functional diversity could be attributed to variations in plant litter quality and substrate inputs to the soil. The abundant resources and fast nutrient turnover in residue retention treatments might contribute to the changes in microbial functional diversity [21], and suggested that the microbial decomposition pathway is relatively more important in the presence than in the absence of crop residues. In this study, the levels of carbohydrate and amino acids utilization (PC1 values), including D-Mannitol, α-D-Glucose and L-Methyl-D-glucoside, were higher with crop residues retention treatments. D-Mannitol constitutes half of the hemicellulose monomer, which is the major constituent of plant cell walls, widely distributed in plant residues of natural ecosystems. Therefore, the results showed that the rhizosphere soil microbial functional diversity were all enhanced by retaining crop residues managements, indicating that a link between soil nutrient cycling and soil microbial community function exists. However, the mechanism by which these factors interact requires further study.

**Conclusion**

The results indicated that the ability of rhizosphere soil microorganisms to utilize carbon source were increased by application of crop residues practices regardless of soil tillage managements. And the ability of rhizosphere soil microorganisms to utilize carbon source with CT and RT treatments were higher than that of NT and RTO treatments. It were conducive to maintain the diversity of microbial community in rhizosphere soil of paddy field by combining application of conventional tillage, rotary tillage with crop residues managements. The species richness and evenness of microbial community in rhizosphere soil were increased with CT and RT treatments. PCA results showed that there were significant differences in carbon substrate utilization patterns among different soil tillage treatments, the variability of rhizosphere soil microbial community were increased with CT and RT treatments. Carbohydrates and amino acids were the main carbon resources utilized by rhizosphere microbes with different soil tillage treatments. Therefore, the results indicated that the diversity and activity of rhizosphere soil microorganisms were improved by combined application of conventional tillage, rotary tillage with crop residues managements, which is the most beneficial soil management to contribute to sustainable agriculture on the double-cropping paddy field of southern China.

**Acknowledgements**

This study was supported by the National Natural Science Foundation of China (31872851), the Innovative Research Groups of the Natural Science Foundation of Hunan Province (2019JJ10003).

**References**

1. Thomas GA, Dalal RC, Standley J. (2007) No-till effects on organic matter, pH, cation exchange capacity and nutrient distribution in a Luvisol in the semi-arid subtropics. *Soil Till Res* 94: 295–304.

2. Rahman MH, Okubo A, Sugiyama S, Mayland HF. (2008) Physical, chemical and microbiological properties of an Andisol as related to land use and tillage practice. *Soil Till Res* 101:10–19.

3. Yang Q, Wang X, Shen Y, Philp JNM. (2013) Functional diversity of soil microbial communities in response to tillage and crop residue retention in an eroded Loess soil. *Soil Sci Plant Nutr* 59: 311–321.

4.Dolan MS, Clapp CE, Allmaras RR, Baker JM. (2006) Soil organic carbon and nitrogen in a Minnesota soil as related to tillage, residue and nitrogen management. *Soil Till Res* 89: 221–231.

5. Jacobs A, Rauber R, Ludwing B. (2009) Impact of reduced tillage on carbon and nitrogen storage of two Haplic Luvisols after 40 years. *Soil Till Res* 102:158–164.

6. Larsen E, Grossman J, Edgell J, Hoyt G. (2014) Soil biological properties, soil losses and corn yield in long-term organic and conventional farming systems. *Soil Till Res* 139: 37–45.

7. Hartmann M, Frey B, Mayer J, Maeder P. (2015) Distinct soil microbial diversity under long-term organic and conventional farming. *ISME J* 9: 1177–1194.

8. Ling N, Zhu C, Xue C, Chen H. (2016) Insight into how organic amendments can shape the soil microbiome in long-term field experiments as revealed by network analysis. *Soil Biol Biochem* 99: 137–149.

9. González-Chávez MA, Aitkenhead-Peterson JA, Gentry TJ, Zuberer D. (2010) Soil microbial community, C, N, and P responses to long-term tillage and crop rotation. *Soil Till Res* 106: 285–293.

10. Nautiyal CS, Chauhan PS, Bhatia CR. (2010) Changes in soil physico-chemical properties and microbial functional diversity due to 14 years of conversion of grassland to organic agriculture in semi-arid agroecosystem. *Soil Till Res* 109: 55–60.

11. Yang XY, Ren WD, Sun BH, Zhang SL. (2012) Effects of contrasting soil management regimes on total and labile soil organic carbon fractions in a loess soil in China. *Geoderma* 177–178: 49–56.

12. Chen ZD, Ti FS, Chen F. (2017) [Soil aggregates response to tillage and residue management in a double paddy rice soil of the Southern China](http://xueshu.baidu.com/s?wd=paperuri%3A(6dbb24e6cf107a53c4ede4cc9d16f2e0)&filter=sc_long_sign&tn=SE_xueshusource_2kduw22v&sc_vurl=http%3A%2F%2Flink.springer.com%2F10.1007%2Fs10705-017-9864-8&ie=utf-8&sc_us=5288800193616488951). *Nutr Cycl Agroecosys* 109: 103–114.

13. Garland JL, Mills AL. (1991) Classification and characterization of heterotrophic microbial communities on the basis of patterns on community-level, sole-carbon-source utilization. *Appl Environ Microbiol* 57: 2351–2359.

14. SAS. (2008) SAS Software of the SAS System for Windows. SAS Institute Inc, Cary, NC, USA.

15. [Weber KP](http://med.wanfangdata.com.cn/Paper/Search?q=作者%3A(Weber+KP)), [Grove JA](http://med.wanfangdata.com.cn/Paper/Search?q=作者%3A(Grove+JA)), [Gehder M](http://med.wanfangdata.com.cn/Paper/Search?q=作者%3A(Gehder+M)), [Anderson WA](http://med.wanfangdata.com.cn/Paper/Search?q=作者%3A(Anderson+WA)). (2013) Data transformations in the analysis of community-level substrate utilization data from microplates. *J Microbiol Meth* 69:461–469.

16. Wang Y, Li CY, Tu C, Hoyt GD. (2017) Long-term no-tillage and organic input management enhanced the diversity and stability of soil microbial community. *Sci Total Environ* 609: 341–347.

17. Zhang X, Ma L, Gilliam SF, Wang Q. (2012) Effects of raised-bed planting for enhanced summer maize yield on rhizosphere soil microbial functional groups and enzyme activity in Henan Province, China. *Field Crop Res* 130: 28–37.

18. Mendham DS, O’Connell AM, Grove TS, Rance SJ. (2003) Residue management effects on soil carbon and nutrient contents and growth of second rotation eucalypts. *Forest Ecol Manage* 181: 357–372.

19. O’Connell AM, Grove TS, Mendham DS, Rance SJ. (2004) Impact of harvest residue management on soil nitrogen dynamics in Eucalyptus globulus plantations in southwestern Australia. *Soil Biol Biochem* 36: 39–48.

20. Entry JA, Fuhrmann JJ, Sojka RE, Shewmaker GE. (2004) Influence of irrigated agriculture on soil carbon and microbial community structure. *Environ Manage* 33: 363–373.

21. van Bruggen AHC, Semenov AM, van Diepeningen AD, de Vos OJ. (2006) Relation between soil health, wave-like fluctuations in microbial populations, and soilborne plant disease management. *Eur J Plant Pathol* 115: 105–122.

22. Hu S, Coleman DC, Hendrix PF, Beare MH. (1995) Biotic manipulation effects on soil carbohydrates and microbial biomass in a cultivation soil. *Soil Biol Biochem* 27: 1127–1135.

23. Mager DM. (2010) Carbohydrates in cyanobacterial soil crusts as a source of carbon in the southwest Kalahari, Botswana. *Soil Biol Biochem* 42: 313–318.

24. Myers RT, Zak DR, White DC, Peacock A. (2001) Land scape level patterns of microbial community composition and substrate use in upland forest ecosystems. *Soil Sci Soc Am J* 65: 359–367.

**Figure legend**

Fig. 1 AWCD changes with incubation progress with different soil tillage treatments at early rice and later rice maturity stages

CT: conventional tillage with residue incorporation; RT: rotary tillage with residue incorporation; NT: no tillage with residue retention; RTO: rotary tillage with residue removed.

The same as below.

Fig. 2 Principal components analysis for carbon utilization of rhizospheric soil microbial communities with different soil tillage treatments at early and late rice maturity stages

Fig. 3 PCA of different carbons sources of rhizosphere soil microbial diversity with different soil tillage treatments (120 h)

A is indicate carbohydrates, B is indicate amino acids, C is indicate carboxylic acids, D is indicate nucleosides.
