# Supplementary material for: Functional diversity of rhizosphere soil microbial communities in response to different tillage and crop residue retention in a double-cropping rice field
Source: PLoS One. 2020 May 21;15(5):e0233642. doi: 10.1371/journal.pone.0233642 (PMC7241825; doi:10.1371/journal.pone.0233642)
Supplement: Attachment — Submitted filename: Response to Reviewers.doc [file pone.0233642.s002.doc]

< PLOS ONE >

< PONE-D-20-02372>

**Dear Editor,**

Thank you very much for your useful comments and suggestions on our manuscript. Please convey our gratitude to the reviewers who have made useful and detailed suggestions for improvement of the manuscript. As their suggestions we have revised the language and the content with red color in the manuscript. The detail of the changes were listed below in point form:

**Reviewer #1:**

1. Page 8 line 10, “significantly differences” should be statistically tested.

√ Page 8 line 10, the “significantly differences” were replaced by “obvious differences”.

2.Page 9 line 11, microbial activity and functional diversity could not be used as the indexes of soil biological fertility.

√ Page 9 line 11, the “biological fertility” were replaced by “microbial community functional diversity”.

3. Page 11 Line 19, please provide evidence from experiments and literatures to support your conclusion that combined application of conventional tillage, rotary tillage with crop residue managements is the most beneficial soil management.

√Page 11 Line 19, these sentences were revised, according to reviewer suggestion.

**Reviewer #2:**

Introduction
1. P3, Line 1-6. This paragraph seems to be not completed. The authors compare two types of agriculture practices: the traditional practice of intensive cultivation and the conservation agriculture and point out that the conservation practice may be the better choice. But afterwards, the authors do not put forward their scientific question: how to effectively evaluate the soil quality under different agriculture regimes?

√ P3, Line 1-6, some sentence about the change of soil quality under different agriculture regimes were added, according to reviewer suggestion.

Methods:
1. P4, Line 25-29. In this part, what are the residues for Chinese milk vetch if the aboveground is harvested? Maybe it is better to not use ‘harvest’ here.

√ P4, Line 25-29, some part of Chinese milk vetch residues were returning to paddy filed after Chinese milk vetch harvested, and the quantity of Chinese milk vetch residues returning to paddy filed for the CT, RT and NT treatments were 22500 kg hm^-2^. Meanwhile, this sentence was revised, according to reviewer suggestion.

2. P4, Line 29-30. There is also confusion here. Why were the residues removed for the treatments of CT, RT and NT?

√ P4, Line 29-30, the total quantity of Chinese milk vetch, early and late rice straw residues for the CT, RT and NT treatments were 52000, 5400, 6000 kg hm^-2^ both the Chinese milk vetch, early and late rice crops were harvested, respectively. On the one hand, according to the local production habits and the optimum returning amount of Chinese milk vetch, early and late rice straw residues, the quantity of Chinese milk vetch, early and late rice straw residues returning to paddy soil for the CT, RT and NT treatments were 22500, 2000, and 2000 kg hm^-2^, respectively. On the other hand, the redundant crop residue need remove from the paddy filed. This is, the other quantity of Chinese milk vetch, early and late rice straw residues removed from the paddy soil for the CT, RT and NT treatments were 29500, 3400, and 4000 kg hm^-2^, respectively.

3. P5, Line 17. It would be better to emphasize that the four treatments were continued in the next three years (2016, 2017 and 2018)

√ P5, Line 17, this sentence were revised, according to reviewer suggestion.

4. P6, Line 27-28. the authors state that ‘The most commonly used methods are correlation analysis and regression analysis.’, but no results from any regression analysis are found in the manuscript.

√ P6, Line 27-28, the sentence of “The most commonly used methods are correlation analysis and regression analysis.” were deleted due to no results from any regression analysis are found in the manuscript, according to reviewer suggestion.

Results:
1.It would be better to present the results of one-way ANOVA of the effects of different tillage treatments on genetic diversity indices of rhizospheric soil microbial communities (F values and p value, etc).

√ The information about the p value of the effects of different tillage treatments on genetic diversity indices of rhizospheric soil microbial communities were added, according to reviewer suggestion.

2.P7, Line 5-7. The first two sentences should moved to the Discussion part.

√ P7, Line 5-7, the first two sentences were moved to the “Discussion ” part, according to reviewer suggestion.

3.P7. Line7-13. It seems that there are no statistics for the AWCD. There are no standard deviation or error for each data point in the Figure 1.The authors also did not present results of any statistical comparison between the four treatments. Furthermore, the authors state that ‘The changing rate of AWCD reflects the ability of soil microorganisms to utilize carbon source’. It would be better to compare the changing rate of AWCD of the four treatments.

√ P7. Line7-13, the standard error for each data point were added in the Figure 1. Meanwhile, the information about statistical compare the changing rate of AWCD with different tillage treatments were added, according to reviewer suggestion.

Discussion.
1.P9. Line 15-18. There is a contradictory statement in ‘This conclusion was further reinforced by the similar performance of the crop residues removal treatments with and without tillage, indicating that under these soils and management conditions tillage was significant influence on soil functionality microbial ecology’. If the performance of the crop residues removal treatments with and without tillage were similar, how can the tillage impose significant effects on soil functionality microbial ecology?

√ P9. Line 15-18, these sentences were revised, according to reviewer suggestion.

Conclusion
1. P11. Line 6-20. The results of the experiment are repeated in this part. Please only summarize all the important findings and their implications.

√P11. Line 6-20, in the “conclusion” section, the structure of this part were revised, some sentences were revised, and the repeated information were deleted, according to reviewer suggestion.

Language
There are many English grammar mistakes. Please correct them. Some of the examples are:
1. Introduction, P3, Line 1: ’ploughings and complete removal of crop residues has resulted in soils low in organic matter, of poor fertility and very susceptible to erosion.’ is changed to ‘ploughings and complete removal of crop residues have resulted in low organic mater content, low fertility and high susceptibility to erosion in soils ’

√Introduction, P3, Line 1, these sentences were revised, according to reviewer suggestion.

2. P3, Line 28: ‘the effect’ changed to ‘the effects’

√ P3, Line 28, the “the effect” were replaced by “the effects”, according to reviewer suggestion.

3. P7 Line 9. ‘There is not change in AWCD with different soil tillage treatments at the beginning of 24 h,’ is changed to ‘There were no changes in AWCD with different soil tillage treatments at the beginning of 24 h,’

√ P7 Line 9, the “There is not change in AWCD with different soil tillage treatments at the beginning of 24 h” were replaced by “There were no changes in AWCD with different soil tillage treatments at the beginning of 24 h”, according to reviewer suggestion.

4. P8. Line 5-11. ‘were mainly distributes’ is changed to ‘were distributed’.

√ P8. Line 5-11, the “were mainly distributes” were replaced by “were distributed”, according to reviewer suggestion.

5. Figure 3 ‘A is indicate carbohydrates, B is indicate amino acids, C is indicate carboxylic acids, D is indicate nucleosides. ’ is changed to ‘A indicates carbohydrates, B indicates amino acids, C indicates carboxylic acids, D indicates nucleosides. ’

√ In Figure 3, the “A is indicate carbohydrates, B is indicate amino acids, C is indicate carboxylic acids, D is indicate nucleosides.” were replaced by “A indicates carbohydrates, B indicates amino acids, C indicates carboxylic acids, D indicates nucleosides.”, according to reviewer suggestion.

**Reviewer #3:**

1.The tense of this manuscript should be consistent.

√ The tense of this manuscript were revised, and keep the tense consistent of this manuscript, according to reviewer suggestion.

2. Data analysis part should be clearer, and some content of result should be removed to this part.

√ The data analysis part were revised, and some content of result were removed to this part, according to reviewer suggestion.

3. Delete Results content in conclusion.

√ In the “conclusion” section, some results content were deleted, according to reviewer suggestion.

4. Add new publications in recent 3 yrs into References.

√ In the “References” section, some references about of new publications in recent 3 year were added, and some old references were replaced by new references, according to reviewer suggestion.

**Reviewer #4:**

1. The abstract are in poor organization. For example, the sentences from lns 8-15 in page 2 should be combined and elucidate in clear words. Such as, four treatments (CT, RT, NT, and RTO) were conducted in the field to investigate the average well color development, richness and evenness of the functional diversity, the substrate reaction. Also, the sentence ln 20 recommended to be rewritten as “…..with the order as…..”

√ In the abstract section, P2, Line 8-15, Line 20, these sentences were revised, according to reviewer suggestion.

2. The introduction should be logically organized by explaining why the soil tillage or crop residues can affect the soil microbes. And then it is concluded that the interactive effects of tillage and crop residues can increase or decrease the soil microbial community diversity. There are many grammatical mistakes in this section. Such as lns 7-8 in page 3, delete the word “in” before nutrient cycling and altering the availability….

√ In the introduction section, the introduction were logically organized, some more information about of the soil tillage or crop residues can affect the soil microbes were added, according to reviewer suggestion.

√ Many grammatical mistakes were revised, such as, lns 7-8 in page 3 according to reviewer suggestion.

3. It is not clear about the experiment design. Especially, how to explore or manipulate the tillage and crop residues addition.

Also, it is not clear about the bacterial community analysis using the Biolog ECO microplates.

Actually, soil tillage and crop residues are two factors in this experiment, and it should be proper to use two-way ANOVA but not the one-way ANOVA.

√ The manipulate the tillage were introduced in the “Field experiment” section, and the manipulate the crop residues addition were also added in the “Field experiment” section.

√ In the “Soil analysis” section, some more detail information about the bacterial community analysis using the Biolog ECO microplates were added, according to reviewer suggestion.

√ These sentences were revised. In the present study, our proceed on a one-way anova to account for different tillage treatments at the maturity stages of early rice and late rice, respectively. And the CT, RT and NT treatments under the same crop residue conditions, that is, the difference of each investigate items between tillage treatments at the same stage of rice were analysis.

4. About the result section, it should state the main findings or results not introduce the meaning of the variables. Such as the sentences lns 5-7, lns 23-25 and the identical sentences in page 7-8.

√ In the “result” section, some sentences of lns 5-7, lns 23-25 in page 7-8 were removed to the other places, according to reviewer suggestion.

5. Adherent to the data, the discussion section should clearly and logically explain the results and compared them the other results.

√ In the “Discussion” section, the organization were revised, that is, the discussion section were clearly and logically explain the results of this experiment. Meanwhile, the reason of some results were added, and some references were added to support these conclusion.

6. The English in the paper needs much improvement.

√ The English grammar of this paper were revised, according to reviewer suggestion.

**Journal Requirements:**

1. Please ensure that your manuscript meets PLOS ONE's style requirements, including those for file naming.

√ The “key words” section were deleted, the format of this manuscript were revised, such as the format of figures, tables and references were modified, and so on, according to Journal Requirements suggestion.

2. We noticed you have some minor occurrence of overlapping text with the following previous publication(s), which needs to be addressed:

-<https://www.tandfonline.com/doi/abs/10.1080/09064710.2019.1662082?src=recsys&journalCode=sagb20>

-https://www.sciencedirect.com/science/article/pii/S0048969717317564?via%3Dihub

In your revision ensure you quote or rephrase any duplicated text outside the methods section.

√ Two references were added and quoted in the revised manuscript, according to Journal Requirements suggestion. That is, the references about “8. Tang HM, Li C, Xiao XP, Tang WG, Cheng KK, Pan XC, et al. Soil physical and chemical quality as influenced by soil tillage managements under double cropping rice system of southern China. Acta Agr Scand B-S P. 2020; 70: 14–23. 14. Wang Y, Li CY, Tu C, Hoyt GD, Deforest JL, Hu SJ. Long-term no-tillage and organic input management enhanced the diversity and stability of soil microbial community. Sci Total Environ. 2017; 609: 341–347.” were added.

3. Thank you for stating the following in the Acknowledgments Section of your manuscript: "This study was supported by the National Natural Science Foundation of China (31872851), the Innovative Research Groups of the Natural Science Foundation of Hunan Province (2019JJ10003)."

We note that you have provided funding information that is not currently declared in your Funding Statement. However, funding information should not appear in the Acknowledgments section or other areas of your manuscript. We will only publish funding information present in the Funding Statement section of the online submission form.

Please remove any funding-related text from the manuscript and let us know how you would like to update your Funding Statement.

√ The funding-related text were removed from the manuscript, and the funding-related text were update in our Funding Statement. That is, the information about “This study was supported by the National Natural Science Foundation of China (31872851), the Innovative Research Groups of the Natural Science Foundation of Hunan Province (2019JJ10003), and Hunan Natural Science Foundation of China (2018JJ3305).” were added in our Funding Statement.

4. PLOS requires an ORCID iD for the corresponding author in Editorial Manager on papers submitted after December 6th, 2016. Please ensure that you have an ORCID iD and that it is validated in Editorial Manager. To do this, go to ‘Update my Information’ (in the upper left-hand corner of the main menu), and click on the Fetch/Validate link next to the ORCID field. This will take you to the ORCID site and allow you to create a new iD or authenticate a pre-existing iD in Editorial Manager.

√ The ORCID iD were validated in Editorial Manager.

5. Please include your tables as part of your main manuscript and remove the individual files. Please note that supplementary tables (should remain/ be uploaded) as separate "supporting information" files.

√ The tables were added in the revised manuscript, according to Journal Requirements suggestion.

**In the other aspect：**

1. The “soil tillage” were replaced by “tillage”.

2. The “crop residues” were replaced by “crop residue”.

3. The “paddy fields” were replaced by “paddy field”.

4. The “the” of some places in this manuscript were deleted.

The revised manuscript has been submitted to your journal. Once again, thank you for your help and support during the process of the improvement of the manuscript and we look forward to your positive response.

Yours sincerely,

Hai-ming Tang
