# Supplementary material for: Functional diversity of rhizosphere soil microbial communities in response to different tillage and crop residue retention in a double-cropping rice field
Source: PLoS One. 2020 May 21;15(5):e0233642. doi: 10.1371/journal.pone.0233642 (PMC7241825; doi:10.1371/journal.pone.0233642)
Supplement: Attachment — Submitted filename: Response to Reviewers.doc [file pone.0233642.s003.doc]

< PLOS ONE >

< PONE-D-20-02372>

**Dear Editor,**

Thank you very much for your useful comments and suggestions on our manuscript. Please convey our gratitude to the reviewers who have made useful and detailed suggestions for improvement of the manuscript. As their suggestions we have revised the language and the content with red color in the manuscript. The details of these changes were listed below in point form:

**Reviewer #2:**

1．P 2 Line 9：there is no explanation of what are CT, RT, NT, and RTO in the abstract.

√ In the “Abstract” section, the information about CT, RT, NT, and RTO were added, according to reviewer suggestion.

2．P 2 Line 8-11. ‘the average well color development (AWCD), richness and evenness of the functional diversity, the substrate reaction.’ This description gives too many details of quantifying functional diversity of rhizosphere soil microbial communities. Please only show the most important one.

√ In the “Abstract” section, this sentence were revised, according to reviewer suggestion.

3．P 2 A general scientific question is still missing here in the first paragraph in the Introduction.

√ In the first paragraph of “Abstract” section, a general scientific question were added, according to reviewer suggestion.

4．P 4-5. It would be better to present the four different treatments by a figure or a table, which can make it more easily understood by readers.

√ In the “Field experiment” section, it is difficult to layout a table to present the different information about four different treatments when I try to present the four different treatments by a table, and the relative information about four different treatments were well described in the present status. Therefore, I think that there is need not to present the four different treatments by a a table.

5．P7. Line 2-6. More details of how the data analysis was performed should be provided.

√In P7. Line 2-6, more information about data analysis were added and revised, according to reviewer suggestion.

6．P7. Line 9-17. P value of the differences should be provided.

√In P7. Line 9-17, P value of the differences were added, according to reviewer suggestion.

7．P 11, Line 15-17. Revise this sentence: ‘The results provided a reasonable hypothesis that enhanced soil conditions for increased rhizosphere soil microbial community function contribute due to the application of crop residue practices [22]’.

√In P 11, Line 15-17, this sentence were revised, according to reviewer suggestion.

8．P 11, Line 20-22. Revise this sentence: ‘The reason maybe that crop residue retention with tillage treatments benefits the soil by increasing the soil environment, moderating moisture and temperature variation across seasons and has been reported favorable to microbial growth in rhizosphere soil [1, 13].’

√In P 11, Line 20-22, this sentence were revised, according to reviewer suggestion.

9．P 11, Line 26-30. Revise this sentence: ‘suggesting that structure of the rhizosphere soil microbial communities may be altered by these two managements, the reason maybe that concentrations of SOC were significantly higher in the crop residue application treatments [14, 23], and soil ecological environment were also increased [8], which provided carbon nutrient and environment for soil microbial multiply [1]’.

√In P 11, Line 26-30, these sentences were revised, according to reviewer suggestion.

**Reviewer #4:**

1. Some sentences in the introduction are not clear. Such as Page 3 line 1 rewritten the sentence “ to increased”. Page 3 lines 9-10 “ Frasier et al. results showed that….” is recommended to be replaced by “Frasier et al. found that ”. also Page 3 lines 11-12 “Yang et al. results showed that….”, of which what is the meaning of “most carbon sources of microbial ”. some other similar errors such as “ results showed that or results indicated that….” should be reorganized.

√In P 3, Line 1, the “ to increased” were replaced by “increased”, according to reviewer suggestion.

√In P 3 lines 9-10, the “results showed that” were replaced by “found that”, according to reviewer suggestion.

√In P 3 lines 11-12, the “indicated” were replaced by “found”, and this sentence were revised, according to reviewer suggestion.

√Other similar errors were also revised, according to reviewer suggestion.

2. In the M & M part, some sentences about the description of study site is somehow tedious and not clear. Such as “this study was carried out on private land , the owner were gave permission to ”. By the way, gave references about the climate of the study site. Rewritten the sentences such as page 5 line 29 “ the applied kinds of were including ” as “the fertilized includes urea, ordinary ”. Some sentences about the soil sample is not clear. For example, “plant roots were removed by passing the samples through a 2-mm mesh sieve”, actually, the size of mesh sieve just can remove large stone or large plant roots in the soil.

√In the M & M part, P4 lines 11-12, this sentence were revised, according to reviewer suggestion.

√In P4 lines 13-15, the reference about the climate of this study site were added, according to reviewer suggestion.

√In P5 line 29, this sentence were revised, according to reviewer suggestion.

√In P6 line 17, this sentence were revised, according to reviewer suggestion.

3. Still, the statistical analysis process is not clear. PCA is a method and is not a kind of parameters or varialbes.

√In the “Statistical analysis” section, this sentence were revised, according to reviewer suggestion.

4. In the discussion, some sentences are not easy to understand. Such as Page 11 lines 9-10 “ provide a reasonable hypothesis that for increased ”; Page 11 line 22 “ has been reported favorable ”; also, more words like “the reason maybe…”will be boring to authors.

√In P11 lines 9-10, this sentence were revised, according to reviewer suggestion.

√In P11 lines 22, these sentences were also revised, according to reviewer suggestion.

5. It is suggested that invite native English speaker or academic researcher to polish the language throughout the whole manuscript.

√ The language of this manuscript were revised by academic researcher, according to reviewer suggestion. Such as the “managements” were replaced by “management”, the “and” were replaced by “with”, and the “the” in some places were deleted, and so on.

The revised manuscript has been submitted to your journal. Once again, thank you for your help and support during the process of the improvement of the manuscript and we look forward to your positive response.

Yours sincerely,

Hai-ming Tang
