# Supplementary material for: Functional diversity of rhizosphere soil microbial communities in response to different tillage and crop residue retention in a double-cropping rice field
Source: PLoS One. 2020 May 21;15(5):e0233642. doi: 10.1371/journal.pone.0233642 (PMC7241825; doi:10.1371/journal.pone.0233642)
Supplement: Attachment — Submitted filename: Response to Reviewers.doc [file pone.0233642.s004.doc]

< PLOS ONE >

< PONE-D-20-02372>

**Dear Editor,**

Thank you very much for your useful comments and suggestions on our manuscript. Please convey our gratitude to the reviewers who have made useful and detailed suggestions for improvement of the manuscript. As their suggestions we have revised the language and the content with red color in the manuscript. The details of these changes were listed below in point form:

**Reviewer #4:**

1. In the abstract section, some sentences are not clear, such as Page 2 lines 8~13 remerge the two sentences about the description about the treatment code (CT, RT, NT, RTO). Page 2 13~14 suggested to rewritten the sentence as “…the values of AWCD with application of crop residue were higher that of without crop residues.”

√ In Page 2 lines 8~13, these sentences were revised, according to reviewer suggestion.

√ In Page 2 lines 13~14, this sentences were revised, according to reviewer suggestion.

2. In page 3 lines 13-21, the references style in main text is not right, it is suggested to delete the year. Line 21, delete the word “some”. Also, replace “some studies” by “other…”. In lines 27-28, it will be easy to understand to rewritten the sentence as “the manipulation of returning the Chinese milk….to the paddy soil….because it provides….”.

√ In Page 3 lines 13~21, the year of the references were deleted, according to reviewer suggestion.

√ In Page 3 lines 21, the “some studies ” were replaced by “other studies”, according to reviewer suggestion.

√ In Page 3 lines 27-28, this sentences were revised, according to reviewer suggestion.

3. In the M& M section, somehow it will be better to rewritten the sentences in page 4 lines 15-18 as “In 2015, the experiment was conducted in Ning Xiang County….China, where is the main area of double-cropping rice and there were no endangered or protected species involved”. Still, there are other mistakes about the sentences, read through this section and polish the language.

√ In the M& M section, in page 4 lines 15-18, these sentences were revised, according to reviewer suggestion. And other places were also revised, according to reviewer suggestion.

4. Although there are improvements about the description of statistical analysis, the description about the PCA is not clear, such as by which software it is performed. It is suggested to reorganized the sentences page 7 lines 10~12.

√ In Page 7 lines 10-12, these sentences were revised, according to reviewer suggestion.

5. Still, it is suggested the author to read through the whole manuscript and reduce the grammar or language mistakes.

√ The grammar or language mistakes of this manuscript were revised, according to reviewer suggestion.

The revised manuscript has been submitted to your journal. Once again, thank you for your help and support during the process of the improvement of the manuscript and we look forward to your positive response.

Yours sincerely,

Hai-ming Tang
